# Muscle Strength Moderates the Relationship between Nutritional Health Risk and Depression in Korean Older Adults

**DOI:** 10.3390/nu14030665

**Published:** 2022-02-04

**Authors:** Jeonghyeon Kim, Seamon Kang, Haeryun Hong, Hyunsik Kang, Ju-Hyoung Kim, Sang-Koo Woo

**Affiliations:** 1 College of Sport Science, Sungkyunkwan University, Suwon 16419, Korea; zzagkim115@naver.com (J.K.); abtkang2@gmail.com (S.K.); hhr8028@skku.edu (H.H.); 2 Department of Physical Education, Andong National University, Andong 36729, Korea; idno10@naver.com (J.-H.K.); exit@andong.ac.kr (S.-K.W.)

**Keywords:** depression, nutrition, physical fitness, lifestyle risk factors, older adults, Koreans

## Abstract

Background: Little is known about the relationships between muscle strength and nutritional health risk with late-in-life depression. This study aimed to investigate the moderating effect of lower-extremity muscle strength on the relationship between nutritional health risk and depression in Korean older adults. Methods: Data obtained from 5949 women and 3971 men aged ≥ 65 years in the 2020 Korea Longitudinal Study on Aging were used in this study. Exposures included lower-extremity muscle strength and nutritional health risk. Lower-extremity muscle strength was measured with a modified sit-to-stand test. The nutritional health risk was assessed using a screening tool. Depression was defined as a score ≥ 8 points on the Geriatric Depression Scale (GDS). Results: Logistic regression analyses showed that depression was positively associated with nutritional health risk (*p* < 0.001) and inversely associated with lower-extremity muscle strength (*p* < 0.001). A moderation analysis with Andrew Hayes’ PROCESS macro showed a significant moderating effect of lower-extremity muscle strength (β = −0.119; 95% confidence interval, −0.172 to −0.066; *p* < 0.001) on the relationship between nutritional health risk and depression; the weaker was the muscle strength, the steeper was the slope of the GDS score for nutritional health risk. Conclusions: The current findings suggest the need for an intervention targeting both high nutritional risk and weak muscle strength as a therapeutic strategy against depression in Korean older adults.

## 1. Introduction

Depression is a serious mental disorder, affecting 3.8% of the global population, and is a leading cause of disability worldwide, representing an important contributor to the global burden of diseases (https://www.who.int/health-topics/depression/ accessed on 1 November 2021). In Korea, depression has been a major public health concern because of its devastating consequences, such as increased risks of chronic diseases [1], mental disorders [2], suicidal ideation [3], and early death [4]. Using data from the Korean national insurance system between 2011 and 2015, an estimated 2.02% of the total Korean population had pharmaceutically treated depression (PTD), and the total medical cost of PTD was USD 1.331 billion annually [5].

Etiologically, depression is significantly correlated with a cluster of behavioral risk factors, including smoking [6], heavy alcohol consumption [7], inactivity [8], and functional limitations [9]. Together with the abovementioned risk factors, weak muscle strength has been used as an important sign of overall health [10], vulnerability [11], fall risk [12], and early death from all causes [13] in older adults. Weak muscle strength is also associated with an increased risk of late-in-life depression in Western [10] and Asian [14] older adults.

Certain components of nutritional status, such as nutritional risk or malnourishment, are other independent risk factors to be considered in relation to the etiology of late-in-life depression. For example, a diet low in fruits and vegetables is associated with an increased risk of depression [15], while a healthy diet, including quality foods, no meat or dairy intake, and vitamin D and omega-3 supplementation, is associated with a lower incidence of depression [16]. In a case–control study involving depressed patients and age-matched healthy controls, unhealthy dietary patterns were associated with higher risks of depression, while healthy dietary patterns were associated with lower risks [17].

Relationships of depression with weak muscle strength and poor nutritional status have been found in Korean older adults [14,18,19,20]. Nevertheless, the prevalence and incidence of late-in-life depression vary across ages [21], perhaps due to individual variations in modifiable lifestyle risk factors. Normal aging results in declines in food consumption and nutritional intake [22,23], as well as reduced muscle mass and strength [24]. In contrast, physical activity and fitness counteract age-related declines [25,26]. Together, findings from previous studies suggest that both weak muscle strength and nutritional health risk are involved in the etiology of late-in-life depression. Therefore, considering both nutrition and muscle strength would provide a better picture of the complex etiology of late-in-life depression.

The current study aimed to investigate the moderating effect of lower-extremity muscle strength on the relationship between nutritional health risk and depression in Korean older adults.

## 2. Materials and Methods

### 2.1. Data Source and Study Participants

**Figure 1:**
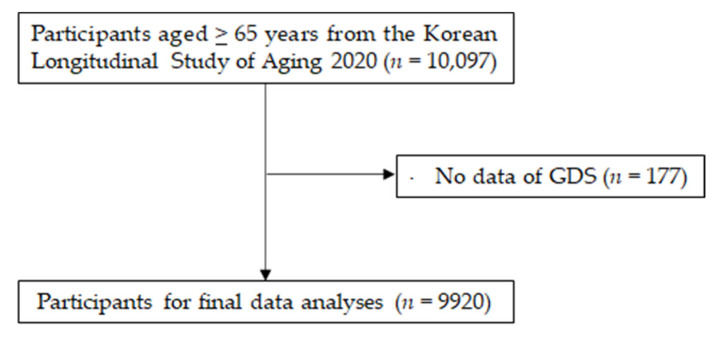
A flowchart for the selection of study participants. GDS; Geriatric Depression Scale.

Data were obtained from the 2020 Korea Longitudinal Study on Aging (KLoSA), a nationwide-population-based survey of 6062 women and 4035 men aged ≥ 65 years conducted in Korea. As shown in Figure 1, those without Geriatric Depression Scale (GDS) data were excluded (*n* = 177). The remaining 9920 samples (including 3971 men and 5949 women) were used for the final data analyses. The Institutional Review Board of the Korea Institute for Health and Social Affairs reviewed and approved the 2020 KLoSA (approval no. 2020-36/approved on 24 March 2020) in accordance with the Declaration of Helsinki. Signed informed consent was obtained from all participants. KLoSA data collection was carried out during face-to-face interviews via a computer-assisted personal interviewing protocol using a tablet. A detailed KLoSA information is accessible via the national public database (https://survey.keis.or.kr/eng/myinfo/login.jsp/ accessed 10 June 2021).

### 2.2. Variables

#### 2.2.1. Depression

Depression was assessed using a Korean version of a 15-item Geriatric Depression Scale (SGDS-K) [27]. Depression was defined as having a score of 8 or higher from the SGDS-K or having physician-diagnosed depression or taking anti-depressant medication(s). The cutoff score of 8 for diagnosing depression was tested and validated in previous studies involving psychiatric patients [28] and community-dwelling elderly persons [27].

#### 2.2.2. Nutritional Health Risk

The nutritional health risk was assessed using the DETERMINE Your Nutritional Health Checklist developed by the Nutrition Screening Initiative [29]. The checklist consists of 14 questions with possible scores of 0 to 4 points. A total score of 0 to 21 points was calculated by summing the values attributed to each question. The nutritional health risk was then categorized as good (0–2), moderate risk (3–5), or high risk (6 or more). The accuracy of the screening tool for nutritional health risk was previously tested and validated in a sample of elderly persons [30].

#### 2.2.3. Lower Extremity Muscle Strength

The sit-to-stand test developed by Buatois et al. [31] was modified to test lower extremity muscle strength. In brief, participants were instructed to stand from a sitting position on a chair or a bed 5 times as quickly as possible while keeping their arms folded across the chest. The performance was scaled according to completeness (1 = completed successfully, 2 = tried but failed to complete, 3 = could not perform). For convenience, “completed successfully” was categorized as indicative of strong muscle strength, and “tried but failed to complete” and “could not perform” were collapsed and categorized as indicative of weak muscle strength.

#### 2.2.4. Covariates

Covariates included age (year), gender (male vs. female), body mass index (BMI) (weight in kg, height in m^2^), educational background (≤elementary, middle/high school, or ≥college), smoking (current/past smoker or nonsmoker), alcohol consumption (0, 1–6, ≥7 times/week), regular exercise (yes or no), and comorbidities. Patients’ comorbidity status was determined using diagnoses reported by a doctor of at least one of 17 selected chronic conditions (i.e., hypertension, stroke, hyperlipidemia, heart disease, kidney disease, diabetes, cancer, hypo- or hyperthyroidism, arthritis, osteoporosis, pain/sciatica in the lower back, fracture/dislocation/accident-related residual injuries, chronic lung disease, asthma, mycobacteria/tuberculosis, dementia, and Parkinson’s disease). The number of chronic conditions (comorbidities) was summed and categorized as no health condition (none), one health condition (single), and two or more health conditions (multiple).

### 2.3. Statistics

**Figure 2:**
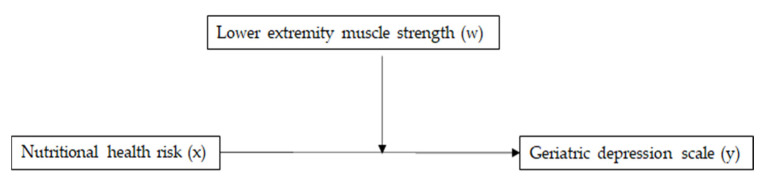
A conceptual diagram for the relationship between nutritional health risk (x) and Geriatric Depression Scale (y) moderated by lower extremity muscle strength (w).

Quantile–quantile plotting and a variance inflation factor were used to verify data distribution normality and the absence of multicollinearity, respectively. Student’s *t*-test and chi-squared test were used to compare continuous and categorical variables, respectively, between not depressed and depressed participants. Linear regression was used to assess the relationships between GDS scores and other variables. Multivariate logistic regression was used to estimate odds ratios (ORs) and 95% confidence intervals (CIs) of nutritional health and lower-extremity muscle strength for depression. Finally, as illustrated in Figure 2, we examined the moderating effect of lower-extremity muscle strength (moderator, W) on the relationship between nutritional health risk (continuous, X) and GDS (continuous, Y) with Andrew Hayes’ PROCESS macro. The statistical significance of the model was tested with bias-corrected bootstrapping (*n* = 5000) and 95% CIs. The statistical significance of a relationship was tested with a nonzero value of a 95% bootstrapped CI. All other statistical significances were tested at *p* = 0.05 using SPSS version 27.0 for Windows (IBM Corporation, Armonk, NY, USA).

## 3. Results

Table 1 presents descriptive statistics of study participants by depression status. Depressed individuals were older (*p <* 0.001) and more likely to be female (*p* = 0.013), with lower mean BMI (*p <* 0.001), lower educational background (*p <* 0.001), weaker lower-extremity muscle strength (*p <* 0.001), and more infrequent regular exercise, greater heavy alcohol consumption (*p =* 0004), more comorbidities (*p <* 0.001), and higher nutritional health risk (*p <* 0.001), compared with nondepressed individuals.

Table 2 presents linear regression analysis results for GDS. GDS was negatively correlated with age (*p <* 0.001), BMI (*p <* 0.001), and lower-extremity muscle strength (*p <* 0.001) but positively with education (*p <* 0.001), multiple comorbidities (*p <* 0.001), and nutritional health risk (*p <* 0.001).

Table 3 presents logistic regression results of nutritional health risk and lower-extremity muscle strength for depression. Moderate (OR, 1.772; 95% CI, 1.472–2.133) and high (OR, 7.703; 95% CI, 6.450–9.199) nutritional health risks were significantly associated with greater odds for depression, compared with good health (OR, 1). The increased odds of moderate (OR, 1.679; 95% CI, 1.385–2.035) and high (OR, 7.571; 95% CI, 6.285–9.119) nutritional health risks for depression remained statistically significant even after adjustments for covariates of age, gender, BMI, education, smoking, alcohol intake, regular exercise, and multiple comorbidities. With respect to lower-extremity muscle strength, weak muscle strength was significantly associated with greater odds for depression (OR, 2.412; 95% CI, 2.060–2.824), compared with strong muscle strength (OR, 1). The increased odds of weak muscle strength for the mental health condition remained statistically significant (OR, 2.094; 95% CI, 1.770–2.476) even after adjustments for all covariates.

**Figure 3:**
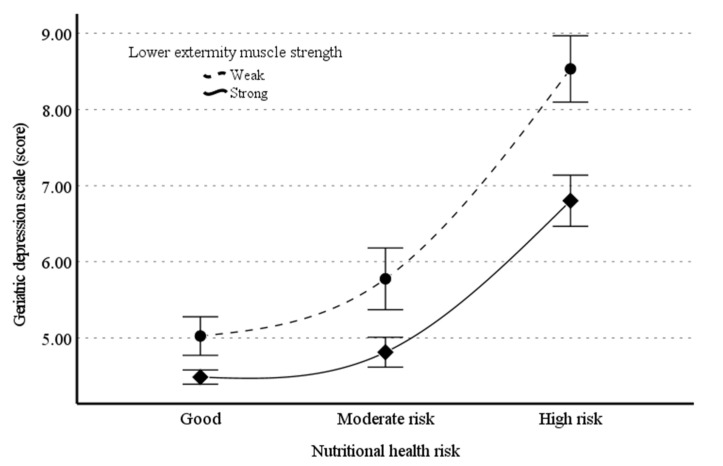
Illustration of the moderating effect of lower extremity muscle strength on the relationship between nutritional health risk and Geriatric Depression Scale.

Table 4 presents the relationship between nutritional health risk (x) and GDS (y) moderated by lower-extremity muscle strength (w). There was a significant moderating effect of lower-extremity muscle strength for the relationship between nutritional health risk and depression (β = −0.127; 95% CI, −0.157 to −0.097). The moderating effect of lower-extremity muscle strength remained significant (β = −0.120; 95% CI, −0.173 to −0.067) after adjustments for the covariates. Additionally, we probed the interaction to better interpret the moderating effect of lower-extremity muscle strength on the relationship between nutritional health risk and GDS; the weaker was the muscle strength, the steeper was the slope of GDS concerning the effect on nutritional health risk (Figure 3).

## 4. Discussion

In this population-based cross-sectional study, we examined the impacts of lower-extremity muscle strength and nutritional health risk on depression in 9920 Korean older adults aged ≥ 65 years and found that nutritional health risk and weak lower-extremity muscle strength were associated with greater odds for late-in-life depression. The novel finding of the current study is that the weaker was the muscle strength, the greater was the impact of nutritional health risk on depression.

The current findings are in accordance with those of previous studies reporting an association between nutritional status and depression in older adults. For example, malnutrition was found to be an independent risk factor for depression in older outpatients in Turkey [32] and community-dwelling older individuals in Bangladesh [33] and China [34]. Poor dietary quality was significantly associated with postpartum depression in a sample of 939 Chinese lactating women [35]. An association between nutritional health risk and depression was also reported by previous studies involving Korean older adults with multiple chronic diseases [36] and community-dwelling Korean older adults [19]. Additionally, food insecurity and poor nutritional status were associated with greater odds for depression in Korean older adults with low incomes [37] and community-dwelling Korean older adults [18], respectively. By contrast, high consumption of fruits, vegetables, low-fat dairy, and antioxidants is inversely associated with depression risk. Additionally, the consumption of specific nutrients, including calcium, chromium, folate, polyunsaturated fatty acids, vitamins, zinc, and magnesium also has antidepressant effects [38,39]. Consequently, the findings of the current study suggest that a healthy diet such as a Mediterranean diet should be recommended as a therapeutic approach against late-life depression.

The findings of the current study are also in line with those of previous studies reporting an association between weak muscle strength and depression in older adults. For example, several previous studies using data obtained from the Korea National Health and Nutrition Examination Survey showed that handgrip strength was inversely associated with depression risk and suicidal ideation in Korean middle-aged and older adults [40,41]. In a cross-sectional study involving 562 community-dwelling Korean men and women aged ≥ 65 years, we showed that lower muscle mass and lower muscle function were individually and synergistically associated with greater odds of depressive symptoms [9]. In 1100 Singaporean women aged 45–69 years, Ganasarajah et al. [42] showed that weak muscle strength was significantly associated with greater odds of depression and/or anxiety symptoms. A recent meta-analysis of 33 results obtained from 16 studies effectively summarizes the inverse associations between muscle strength and late-in-life depression risk [43].

In particular, the findings of this study extend those of previous studies by showing that muscle strength is an important moderator in determining the relationship between nutritional health risk and late-in-life depression. The moderating effect of lower-extremity muscle strength on the relationship implies the clinical importance of both good nutrition and muscle fitness as a therapeutic strategy against late-in-life depression. In support of the current findings, Pinheiro et al. [44] conducted a 12-week randomized control trial of sarcopenic elderly women and showed that a combination of nutritional intervention and group-based functional exercise is more effective at relieving depressive symptoms and social isolation, compared with a single intervention, such as nutritional intervention or socialization activities. In a cross-sectional study involving 130 community-dwelling Japanese older adults, Kaburagi et al. [45] found that adequate nutritional status was positively associated with handgrip strength and maximum walking speed but negatively associated with GDS score, implying a pathologic connection between nutrition, physical fitness, and mental health. In this aspect, muscle strength might reflect nutritional health status, or vice versa, which contributes to mental health. However, the exact direction of the relationship cannot be determined due to the cross-sectional nature of the study. 

Some explanations are possible regarding the modulating effect of lower-extremity muscle strength on the relationship between nutritional health risks and late-in-life depression. First, chronic exposure to nutritional health risks might lead to or exacerbate geriatric conditions that contribute to depression, such as functional limitations [46], physical disabilities [47], and sarcopenia [48]. Consequently, the moderating effect of lower-extremity muscle strength on the relationship between nutritional health risk and depression might reflect an intermediate phenotype linking nutritional health risk to mental illness. However, the exact mechanism(s) by which muscle strength attenuates the negative effect of nutritional health risk on mental health must be determined.

Second, maintaining muscle mass and strength itself may minimize or counteract the risk of late-in-life depression [49]. For example, strong muscle strength is associated with physical independence, fewer difficulties performing daily activities, lower risk for experiencing physical disabilities, and better quality of life [50,51]. Furthermore, muscle contraction has an anti-depressant effect by suppressing pro-inflammatory responses (i.e., decreased levels of circulating tumor necrosis factor-α and interleukin 6) while enhancing anti-inflammatory responses (i.e., increased levels of circulating interleukin-10 and brain-derived neurotrophic factor) [52,53].

Third, other health conditions, such as chronic diseases, increased oxidative stress, immobility, and dysregulated hormonal cycles, which are attributed to nutritional health risk and/or weak muscle strength, might be involved in the moderating effect of lower-extremity muscle strength on the relationship between nutritional health risk and depression [49,54].

Lastly, poor socio-economic status (i.e., poverty, living alone, low education, and illiteracy) and poor health behaviors (i.e., smoking and heavy alcohol consumption) may be entangled in the moderating effect of lower extremity muscle strength on the relationship between nutritional health status and late-life depression [55]. The involvement of those risk factors in the interconnection between muscle strength and nutritional health risk on depressive symptoms remains to be investigated in a future study.

This study is not without limitations. First, the cross-sectional nature of this study does not allow us to offer any cause-and-effect interpretation regarding the moderating effect of lower-extremity muscle strength on the relationship between nutritional health risk and depression. Second, the relationships of nutritional health risk and lower-extremity muscle strength with depression can be bidirectional. In other words, either nutritional health risk or lower-extremity muscle strength or both can increase the risk for depression, or vice versa, and this remains to be addressed in a well-designed intervention study. Third, the relationships of nutritional health risk and lower-extremity muscle strength with late-in-life depression may imply adverse effects of other health conditions that are not included in the current study. Consequently, caution is necessary when interpreting the moderating effect of muscle strength on the relationship between nutritional health risk and depression.

Despite these limitations, this study also has strengths. First, it is a population-based study with large sample size. Second, to the best of our knowledge, we are the first to report the potential of lower-extremity muscle strength as an important moderator in determining the relationship between nutritional health risk and depression in older adults.

## 5. Conclusions

In this population-based study, we examined the relationships of nutritional health risk and lower-extremity muscle strength with depression in Korean older adults and found that late-in-life depression was positively associated with nutritional health risk and inversely with lower-extremity muscle strength. Particularly, we found that high lower-extremity muscle strength attenuates the negative impact of nutritional health risk on depression, implying an urgency of an intervention targeting both nutritional health risks and weak muscle strength as a therapeutic strategy against depression in Korean older adults.

## Figures and Tables

**Table 1:** Descriptive statistics of study participants (*n* = 9920).

Variables		Not Depressed (*n* = 9042)	Depressed (*n* = 878)	Effect Size	*p* Value
Age (years)				−0.217	<0.001
	60–69	3246 (35.9)	265 (30.2)		
	70–74	2276 (25.2)	190 (21.6)		
	75–79	1765 (19.5)	191 (21.8)		
	≥80	1755 (19.4)	232 (26.4)		
Gender, *n* (%)				0.025	0.013
	Male	3654 (92.0)	317 (8.0)		
	Female	5388 (90.6)	561 (9.4)		
Body mass index (kg/m^2^)		23.6 ± 2.6	23.1 ± 2.7	0.182	<0.001
Education, *n* (%)				0.058	<0.001
	Elementary or less	3968 (43.9)	463 (52.7)		
	Middle/high	4590 (50.8)	394 (44.9)		
	College or higher	484 (5.4)	21 (2.4)		
Smoking, *n* (%)				0.014	0.154
	Nonsmokers	8062 (89.2)	769 (87.6)		
	Current/past smokers	980 (10.8)	109 (12.4)		
Alcohol intake (times/week)				0.004	0.004
	0	5735 (63.4)	508 (57.9)		
	1–6	3207 (35.5)	361 (41.0)		
	≥7	100 (1.1)	9 (1.0)		
Regular exercise, *n* (%)				0.053	<0.001
	Yes	4802 (53.1)	385 (43.8)		
	No	4240 (46.9)	493 (43.8)		
Multicomorbidity, *n* (%)				0.072	<0.001
	None	1598 (17.7)	80 (9.1)		
	Single	2678 (29.6)	244 (27.8)		
	Multiple	4766 (52.7)	554 (63.1)		
Nutritional health, *n* (%)				0.296	<0.001
	Good	6688 (74.0)	370 (42.1)		
	Moderate risk	1753 (19.4)	190 (21.6)		
	High risk	601 (6.6)	318 (36.2)		
Lower body muscle strength, *n* (%)				0.174	<0.001
	Strong	7588 (84.1)	530 (60.5)		
	Weak	1437 (15.9)	346 (39.5)		

**Table 2:** Linear regression for the determinants of Geriatric Depression Scale.

Variables	β	95% CI	r^2^_part_	*p* Value	VIF
Age	−0.010	−0.018~−0.002	0.005	<0.001	1.475
Gender	−0.003	−0.102~0.097	−0.004	0.510	1.385
Body mass index	−0.032	−0.048~−0.016	−0.041	<0.001	1.024
Education	0.143	0.059~0.227	0.001	<0.001	1.409
Smoking	0.043	−0.101~0.309	−0.013	0.152	1.183
Alcohol intake	0.217	−0.126~0.089	0.051	0.471	1.263
Regular exercise	0.004	−0.081~0.220	0.002	0.271	1.054
Multicomorbidity	0.171	0.290~0.112	0.012	<0.001	1.125
Nutritional health risk	0.275	0.336~0.290	0.354	<0.001	1.103
Lower extremity muscle strength	−0.340	−0.456~−0.224	−0.117	<0.001	1.157

CI: confidence interval; VIF: variance inflation factor.

**Table 3:** Odds ratios (ORs) and 95% confidence intervals (CIs) of depression by nutritional health and lower body strength.

Predictors	Model 1		Model 2	
	OR (95% CI)	*p* Value	OR (95% CI)	*p* Value
Nutritional health risk				
Good	1 (reference)		1 (reference)	
Moderate risk	1.772 (1.472~2.133)	<0.001	1.679 (1.385~2.035)	<0.001
High risk	7.703 (6.450~9.199)	<0.001	7.571 (6.285~9.119)	<0.001
Lower extremity muscle strength				
Strong	1 (reference)		1 (reference)	
Weak	2.412 (2.060~2.824)	<0.001	2.094 (1.770~2.476)	<0.001

Model 1 unadjusted. Model 2 adjusted for age, gender, body mass index, education, smoking, alcohol intake, regular exercise, and multicomorbidity.

**Table 4:** A moderation analysis of lower body strength for the relationship between nutritional health and Geriatric Depression Scale.

Predictors	Coefficients	SE	t	*p*	95% CI	
					Lower	Upper
Model 1 (R^2^ = 0.133, F = 506.561, *p* < 0.001)						
Nutritional health	0.472	0.026	18.064	<0.001	3.736	4.256
Lower body strength	0.110	0.071	1.562	0.118	−0.028	0.249
Interaction	−0.127	0.015	−8.342	<0.001	−0.157	−0.097
R^2^ change due to the moderator = 0.006 (F = 69.59, *p* < 0.001)						
Model 2 (R^2^ = 0.162, F = 71.006, *p* < 0.001)						
Nutritional health	0.494	0.046	10.680	<0.001	0.403	0.584
Lower body strength	−0.356	0.133	−2.673	0.008	−0.617	−0.095
Interaction	−0.120	0.027	−4.451	<0.001	−0.173	−0.067
R^2^ change due to the moderator = 0.005 (F = 19.809, *p* < 0.001)						

Model 1 unadjusted. Model 2 adjusted for age, gender, body mass index, education, smoking, alcohol intake, regular exercise, and multicomorbidity. SE: standard error; CI: confidence interval.

## Data Availability

Data are accessible via the National public database (https://survey.keis.or.kr/eng/myinfo/login.jsp/ accessed 10 June 2021).
